# Everybody's Page

**Published:** 1890-05-17

**Authors:** 


### Everybody's Page.

In the year 1319 sugar is said to have been sold at one shilling and nine pence halfpenny a pound, and was considered a very luxurious article till the eighteenth century.

Over one hundred and sixty million gallons of water are said to be supplied on an average daily to houses in London by the London Water Companies, each house receiving on an average about 200 gallons.

The Ladies' Health Association of New York is a very energetic body, and has accomplished all sorts of reforms in the City during the last few years, notably in the matters of school hygiene, sewers, and slaughter-houses. They hope in a short time to open public crematories in which to consume refuse.

Foreigners often laugh at some of our culinary customs, such as taking apple sauce with roast pork or goose. There is probably not so much incongruity in this mixture as at first sight appears. The acid of ripe apples helps to neutralize the excess of chalky matter which is produced by eating meat in too great quantities.

According to our ideas of a healthful life, a stoker on one of the great ocean steamers ought to suffer considerably, and be shortlived. Yet stokers, as a rule, are very jolly and well, and they scarcely ever catch cold. They work four hours at a time in the great heat, refreshing themselves every now and then at the air-pipe, then they turn in for eight hours. The "grog" has been discontinued in the big English and American liners, as drink was found to be particularly bad for stokers.

There was a lecture not long since at the Chester Society of Natural Science when long dissertations were held on the bad behaviour of the sparrows, who were calculated by one member to do harm to grain in England to the awful extent of five million pounds. It is very difficult to say who is right and who wrong, but this sum sounds a trifle large. No wonder members wanted to exterminate the sparrows! We are inclined to think, however, that the sparrow is like the proverbial household "cat," made to answer for his own and others sins as well.

The Japanese artist, however talented, never ranks higher than an artisan, be he sculptor, painter, worker in enamel, or anything else. So many changes have taken place of recent years in Japan, mainly owing to European influences, that their position will probably be altered for the better before many years elapse. The Japanese are great reformers, and the recent discussion in the leading Japanese newspapers on the position of artists in that country is evidence of a coming change. The comparative obscurity and social neglect of art workers, is alleged by some to be the result of absence of organization, but insufficient remuneration also does not tend to ameliorate their position.

There is a very good story in modern days of gratitude before and after a boon has been granted. A wealthy merchant, having injured his little finger, was told by a doctor that amputation was imperative. This so distressed his vanity that he consulted a celebrated surgeon as to whether he considered such stringent measures necessary. The surgeon promised to save the finger by every other means in his power. At each visit the patient's gratitude increased, and then came the day when he inquired his indebtedness to the doctor. "That sir," said the surgeon, "depends upon what value you set upon your finger." The patient hesitated, and then generously remarked "Well, you see it was only my *little* finger after all."

Those who are interested in the establishment of a Nationa School of Forestry in this country are looking forward to the planting of the 27 million acres of land which they state to be now out of cultivation. But have they taken into consideration the quality of the soil upon which profitable planting will to a great extent depend?

Owing to the prohibition placed on the sale of lead in Upper Burma, with a view to depriving the dacoits of the means of getting bullets, a great quantity of lead has now accumulated at the pit-heads of the lead mines in that country. Here is an opportunity for a remunerative trade as soon as the country is completely pacified.

There seem to be few things in these days which science cannot turn to good account. Apparently sugar of good quality can be made out of sawdust or rags. We must confess we would prefer to believe that the sugar we eat was manufactured out of something more palatable. The *School Newspaper* is responsible for this information.

It is popularly supposed that to have the head raised on a pillow is the proper position in which to sleep, but an American doctor advocates the raising of the feet higher than the head as the only rational position. In support of his view, he claims that the brain is thus better nourished by a more plentiful supply of blood, but not so plentiful as to cause congestion.

Dr. Gilman Thompson has written a paper on the "Fallacy of the So-called Hot Air Treatment of Phthisis," which he says appears to him to be based on false principles altogether, and likely to lead to negative if not injurious results. His conclusions, which are drawn from a series of experiments, are that the continued inhalation of hot air from 200° to more than 300° F., does not, in many instances, raise the temperature of the lung at all. In other cases there might be a small rise from two to four degrees owing to some other causes, while the temperature of the trachea did not increase more than four to six degrees.

The old story of the man who can well afford to pay for medical advice, but who gets it free at the dispensary or hospital offers examples in America as well as in England, and it is a comfort to see that the Free Eye, Ear, Nose, and Throat Hospital, of New Orleans, means to try and stop the abuse somehow or other. For as the report says, "it is our imperative duty to husband our resources in order to relieve the *truly* poor and needy." As by giving gratuitous treatment to those fully able to pay we do "a great injustice to the medical profession, a class of meritorious citizens noted for their liberality and charity." We wish a good many more people could be induced to see things in this light.

The old methods of tobacco smoking must sound rather devoid of comfort to the luxurious man of the present day who revels in as many smokes in the hour as he cares to have. According to Aubrey, silver pipes were first used by rich tobacco smokers, but the ordinary humble mortal used a walnut shell and a straw, which had at least this merit, that a clean straw did away with the danger of nicotine. In those days the pipe went the round of the table; one man took a whiff and then passed it on to the next who sat longing for his turn to come again. Take this to heart excessive consumer of Egyptian cigarettes! and reflect how much better for you if tobacco were as dear and as scarce now as it was in those days.

